# Neutrophil degranulation interconnects over-represented biological processes in atrial fibrillation

**DOI:** 10.1038/s41598-021-82533-5

**Published:** 2021-02-03

**Authors:** Makiri Kawasaki, Eva R. Meulendijks, Nicoline W. E. van den Berg, Fransisca A. Nariswari, Jolien Neefs, Robin Wesselink, Sarah W. E. Baalman, Aldo Jongejan, Tim Schelfhorst, Sander R. Piersma, Thang V. Pham, Wim J. P. van Boven, Antoine H. G. Driessen, Connie R. Jimenez, Joris R. de Groot

**Affiliations:** 1 Department of Clinical and Experimental Cardiology, Amsterdam Cardiovascular Sciences, Amsterdam UMC, University of Amsterdam, Heart Centre, Meibergdreef 9, 1105 AZ Amsterdam, The Netherlands; 2 Department of Clinical Epidemiology, Biostatistics and Bioinformatics, Bioinformatics Laboratory, Amsterdam UMC, University of Amsterdam, Meibergdreef 9, 1105 AZ Amsterdam, The Netherlands; 3 OncoProteomics Laboratory, Amsterdam UMC, Cancer Centre Amsterdam, VU University Medical Centre, De Boelelaan 1117, 1081 HV Amsterdam, The Netherlands

**Keywords:** Cell biology, Cardiology, Diseases, Pathogenesis

## Abstract

Despite our expanding knowledge about the mechanism underlying atrial fibrillation (AF), the interplay between the biological events underlying AF remains incompletely understood. This study aimed to identify the functionally enriched gene-sets in AF and capture their interconnection via pivotal factors, that may drive or be driven by AF. Global abundance of the proteins in the left atrium of AF patients compared to control patients (n = 3/group), and the functionally enriched biological processes in AF were determined by mass-spectrometry and gene set enrichment analysis, respectively. The data were validated in an independent cohort (n = 19–20/group). In AF, the gene-sets of innate immune system, metabolic process, cellular component disassembly and ion homeostasis were up-regulated, while the gene-set of ciliogenesis was down-regulated. The innate immune system was over-represented by neutrophil degranulation, the components of which were extensively shared by other gene-sets altered in AF. In the independent cohort, an activated form of neutrophils was more present in the left atrium of AF patients with the increased gene expression of neutrophil granules. MYH10, required for ciliogenesis, was decreased in the atrial fibroblasts of AF patients. We report the increased neutrophil degranulation appears to play a pivotal role, and affects multiple biological processes altered in AF.

## Introduction

Atrial fibrillation (AF) is the most common sustained arrhythmia characterized by rapid and multiple irregular depolarization within the atria. AF is associated with serious morbidity and increased mortality^1^, and its prevalence is prospected to further increase as society ages^2^. The limited efficacy of treatments to cure AF^3^ as well as its high socioeconomic burden^4^ makes AF a major clinical challenge.

Once AF occurs, it induces an alteration in the expression and function of ion channels referred to as electrical remodelling, which creates a functional substrate for re-entry within atria that facilitates induction and maintenance of AF^5^. When AF episodes last longer, structural remodelling of the atria emerges which, in turn, facilitates the tenacity of AF^6^ and ultimately perpetuation of AF. A variety of biological events play a role in the forementioned pathogenesis and the progression of the AF substrate^7^. Our knowledge on the involvement of individual biological events in the formation of arrhythmogenic substrate such as ageing^8^, obesity^9^, inflammation^10^ and oxidative stress^11^, has enormously increased over the last decades^12^. However, an unbiased overview of proteins and functionally enriched biological processes as well as their crosstalk have not been demonstrated yet.

Explorative proteomics analysis by high-throughput liquid-chromatography online coupled to tandem mass spectrometry (LC–MS/MS) is a strong tool to uncover the changes in global protein abundance along with the altered biological processes in various diseases, and can thereby give insight into disease biology. Several studies have taken this approach to discover novel biomarkers in plasma in AF, or identify proteins with altered abundance within a single biological process such as cardiac metabolism^13^ and atrial fibrosis^14,15^. However, the pathophysiological changes in AF are likely not driven by a single protein, but rather orchestrated by multiple biological processes involving a large number of proteins.

Here, we conducted an exploratory proteomics analysis in left atrial tissue from patients with and without a history of AF to determine the global expression profile of proteins and comprehensively deciphered the altered biological events driving, or driven by AF.

## Results

### Clinical characteristics of the patients

The baseline clinical characteristics of the study population enrolled for the proteomics analysis (non-AF: n = 3, AF: n = 3) and an independent cohort (non-AF: n = 20, AF: n = 19) for the validation study are summarized in Tables 1 and 2, respectively. Age, CHA_2_DS_2_-VASc score and blood leukocytes count in AF patients were numerically lower than in non-AF patients in both the proteomics and the validation cohort (Tables 1 and 2), while the other baseline parameters were not significantly different between the groups.

**Table 1:** Clinical characteristics of the patients enrolled for the proteomics analysis.

	**ALL** **N = 6**	**Non-AF** **#1**	**Non-AF** **#2**	**Non-AF** **#3**			**Average of non-AF**	**AF** **#4**		**AF** **#5**		**AF** **#6**		**Average of AF**	***p-value***
**Surgery type**	*–*	CABG	CABG	CABG			*NA*	VATS PVI		VATS PVI		VATS PVI		*NA*	*NA*
**Baseline**															
Sex, male, n (%)	6 (100)	male	male	male			3 (100)	male		male		male		3 (100)	1.00
Age, years (± SD)	63 ± 8	65	68	70			67.7 ± 2.5	66		47		62		58.3 ± 10	0.19
AF type, persistent, n (%)	*–*	*–*	*–*	*–*			*NA*	1		1		1		3 (100)	*NA*
AF duration, years [IQ]	*–*	0	0	0			0 [0]	3		4		1		3 [1–4]	*NA*
BMI, kg/m^2^ (± SD)	27.8 ± 4	31.3	27.3	25.6			28.0 ± 2.9	30.7		21.0		31.0		27.6 ± 5.7	0.91
Creatinine, μml/l (± SD)	84 ± 12	88	67	97			84 ± 15	76		79		97		84 ± 11	1.00
CHA_2_DS_2_-VASc [IQ]	1.7 ± 1.0	3	2	2			2.3 ± 0.6	1		0		2		1.0 ± 1.0	0.12
Vascular disease, n (%)	5 (83)	1	1	1			3 (100)	1		0		1		2 (67)	1.00
Previous PCI, n (%)	1 (17)	0	0	0			0 (0)	0		0		1		1 (33)	1.00
Myocardial infarction, n (%)	1 (17)	0	0	1			1 (33)	0		0		0		0 (0)	1.00
Hypertension, n (%)	4 (67)	1	1	1			3 (100)	0		0		1		1 (33)	0.40
Diabetes Mellitus, n (%)	2 (33)	1	0	0			1 (33)	0		0		1		1 (33)	1.00
Congestive heart failure, n (%)	0 (0)	0	0	0			0 (0)	0		0		0		0 (0)	1.00
Stroke/TIA/embolus, n (%)	0 (0)	0	0	0			0 (0)	0		0		0		0 (0)	1.00
**Hematology**															
Leukocytes, 10^–9^/L [IQ]	9.0 [10.0–5.5]	13.1	9.0	7.7			9.0 [7.7–13.1]	6.3		6.1		3.5		6.1 [3.5–6.3]	0.07
Thrombocytes, 10^–9^/L [IQ]	311 [314–220]	323	311	229			311 [229–323]	308		253		193		253 [193–308]	0.46
**Medication**															
NOAC, n (%)	3 (50)	0	0	0			0 (0)	1		1		1		3 (100)	*NA*
Antiplatelet, n (%)	3 (50)	1	1	1			3 (100)	0		0		0		0 (0)	*NA*
Carbasalate calcium, n (%)	3 (50)	1	1	1			3 (100)	0		0		0		0 (0)	*NA*
Clopidogrel, n (%)	1 (17)	0	0	1			1 (33)	0		0		0		0 (0)	*NA*
Class IA AAD	0 (0)	0	0	0			0 (0)	0		0		0		0 (0)	1.00
Class IC AAD	0 (0)	0	0	0			0 (0)	0		0		0		0 (0)	1.00
Class II AAD	3 (50)	1	1	0			2 (67)	0		0		1		1 (33)	1.00
Class III AAD	0 (0)	0	0	0			0 (0)	0		0		0		0 (0)	1.00
Class IV AAD	0 (0)	0	0	0			0 (0)	0		0		0		0 (0)	1.00
Digoxin, n (%)	0 (0)	0	0	0			0 (0)	0		0		0		0 (0)	1.00
Statins, n (%)	4 (67)	1	1	1			3 (100)	0		0		1		1 (33)	0.40
NSAIDs, n (%)	0 (0)	0	0	0			0 (0)	0		0		0		0 (0)	*NA*
Steroids, n (%)	0 (0)	0	0	0			0 (0)	0		0		0		0 (0)	*NA*

The cohort for the proteomics analysis contains three non-AF patients (#1, #2, #3) and three persistent AF patients (#4, #5, #6).

*VATS PVI* video-assisted thoracoscopic pulmonary vein isolation, *CABG* coronary artery bypass grafting, *BMI* body mass index, *PCI* Percutaneous coronary intervention, *TIA* transient ischemic attack, *NOAC* non-vitamin K antagonist oral anticoagulants, *AAD* anti-arrhythmic drugs, *NSAIDs* Nonsteroidal anti-inflammatory drugs, *NA* not applicable.

**Table 2:** Clinical characteristics of the patients enrolled for the validation study.

	**All** **n = 39**	**Non-AF** **n = 20**	**AF** **n = 19**	***p-value***
**Surgery type**				
VATS PVI		–	19	
CABG		13 (65)	–	
Aortic valve		4 (20)	–	
CABG + valve		3 (15)	–	
**Baseline**				
Sex, male, n (%)	28 (72)	14 (70)	14 (74)	1.00
Age, years (± SD)	64 ± 6.8	65.8 ± 7.0	62.1 ± 6.1	0.09
AF duration, years [IQ]	–	–	0 [0–5.25]	*NA*
Previous catheter PVI, n (%)	–	–	2 (5)	*NA*
BMI, kg/m^2^ (± SD)	27.9 ± 3.4	28.2 ± 3.5	27.6 ± 3.4	0.63
Creatinine, μml/l (± SD)	85.3 ± 12.9	86.2 ± 15	74.4 ± 11.2	0.68
CRP, mg/L [IQ]	2.7 [0.9–2.4]	1.4 [1.0–2.0]	1.9 [0.78–3.4]	1.00
CHA_2_DS_2_-VASc [IQ]	2 [1–3]	3 [2–3]	1 [1–2]	< 0.01
Vascular disease, n (%)	21 (54)	17 (85)	4 (21)	< 0.01
Previous PCI, n (%)	8 (21)	6 (30)	2 (11)	0.24
Myocardial infarction, n (%)	10 (26)	7 (35)	3 (16)	0.27
Hypertension, n (%)	19 (49)	12 (60)	7 (37)	0.21
Diabetes Mellitus, n (%)	5 (13)	4 (20)	1 (5)	0.34
Congestive heart failure, n (%)	0	0	0	*NA*
Stroke/TIA/embolus, n (%)	5 (13)	4 (20)	1 (5)	0.34
**Hematology**				
Leukocytes, 10^–9^/L [IQ]	6.7 [5.6–8.6]	8.0 [6.0–9.5]	5.7 [4.8–7.5]	< 0.05
Thrombocytes, 10^–9^/L [IQ]	225.5 [205.5–264.3]	241.5 [214–281.3]	206.5 [196.3–258.5]	0.16
**Medication**				
NOAC/vitK antagonist, n (%)	19 (49)	0 (0)	19 (100)	*NA*
Antiplatelet, n (%)	18 (46)	18 (90)	0 (0)	*NA*
Carbasalate calcium	16 (41)	16	0 (0)	*NA*
Clopidogrel	5 (13)	5	0 (0)	*NA*
Ticagrelor	1 (3)	1	0 (0)	*NA*
Class IA AAD, n (%)	0	0	0	*NA*
Class IC AAD, n (%)	3 (8)	0	3 (16)	0.11
Class II AAD, n (%)	22 (57)	13 (65)	9 (47)	0.34
Class III AAD, n (%)	9 (23)	1 (5)	8 (42)	< 0.01
Class IV AAD, n (%)	0	0	0	*NA*
Digoxin, n (%)	4 (10)	0	4 (21)	< 0.05
Statins, n (%)	20 (51)	13 (65)	7 (35)	0.11
NSAIDs, n (%)	2 (5)	1 (5)	1 (5)	*NA*
Steroids, n (%)	0 (0)	0 (0)	0 (0)	*NA*

The cohort for the validation study contains 20 non-AF patients and 19 persistent AF patients.

*VATS PVI* video-assisted thoracoscopic pulmonary vein isolation, *CABG* coronary artery bypass grafting, *BMI* body mass index, *CRP* C-reactive protein, *PCI* Percutaneous coronary intervention, *TIA* transient ischemic attack, *NOAC* non-vitamin K antagonist oral anticoagulants, *AAD* anti-arrhythmic drugs, *NSAIDs* Nonsteroidal anti-inflammatory drugs, *NA* not applicable.

### The comprehensive expression profile of the identified proteins in the left atrial tissue from non-AF and AF patients

In total, 3153 proteins were identified in the six protein samples extracted from the left atrial tissue of the patients. Principal component analysis demonstrated that the expression profile of the identified proteins in each sample relatively separated non-AF patient #1 from the others in the first principal component (PC1), while the two biological groups (non-AF and AF) were distinct in the second principal component (PC2) (Supplementary Figure S2a-b), suggesting that the source of sample variance potentially lies in AF but a major variance is observed among the low number of non-AF patients.

**Figure 1:**
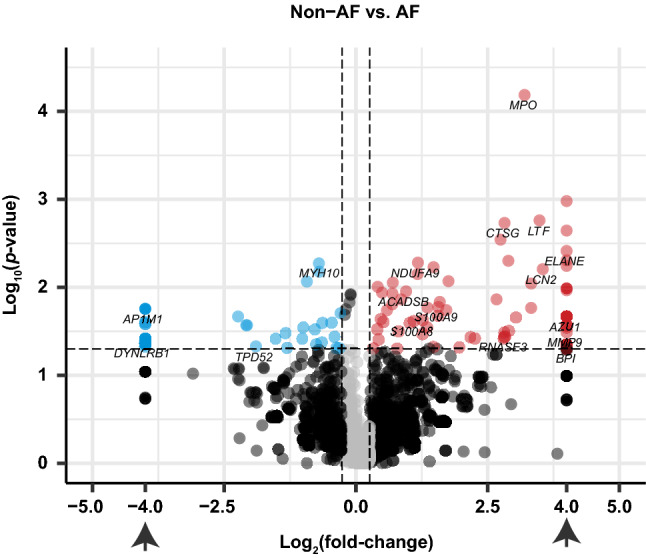
Volcano plot of the 3153 proteins identified by LC–MS/MS. The volcano plot shows the fold-change (x-axis) versus the significance (y-axis) of the identified 3153 proteins. The significance (non-adjusted *p*-value) and the fold-change are converted to −Log_10_(*p*-value) and Log_2_(fold-change), respectively. The vertical and horizontal dotted lines show the cut-off of fold-change =  ± 1.2, and of *p*-value = 0.05, respectively. Those proteins with *N/A* fold-change are plotted on Log_2_(fold-change) =  ± 4 (arrows). There were 115 proteins increased by > 1.2-fold with *p*-value < 0.05 in AF (upper-right, dots colored red), and there were 55 proteins that were decreased by < − 1.2-fold with *p*-value < 0.05, (upper-left, dots colored blue). The volcano plot was generated using R software^54^ (version 3.5.3).

The significance versus fold-change of the identified 3,153 proteins is shown in a volcano plot (Fig. 1). One hundred fifteen proteins (Fig. 1, upper right) and 55 proteins (Fig. 1, upper left) were significantly increased and decreased (non-adjusted *p* < 0.05), respectively, in AF compared to non-AF. Myeloperoxidase (MPO), a peroxidase enzyme released from neutrophil granulocytes^16^, had the most significant change in abundance and was increased 9.2-fold in AF, while myosin heavy chain 10 (MYH10, also known as non-muscle myosin IIB) was most significantly decreased in AF (Supplementary Table 1).

### Altered biological pathways in left atrial tissue of AF patients

**Figure 2:**
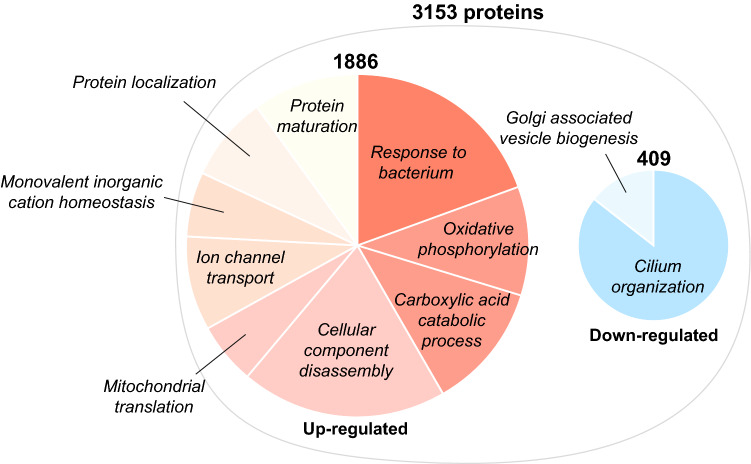
Altered biological pathways in left atrial tissue of AF patient. The result of GSEA is summarized. The right and left pie chart show the up-regulated and down-regulated gene-sets in AF (FDR-adjusted *p*-value < 0.1), respectively. The gene-sets that hierarchically belong to the same/close branch, or share > 40% of the genes are colored together.

Next, we performed Gene Set Enrichment Analysis (GSEA) using a list of all the identified proteins pre-ranked by the significance (*p*-value) considered with biological relevance (fold-change)^17,18^. Overall, 51 gene-sets were significantly overrepresented in AF at the cut-off of FDR-adjusted *p*-value below 0.1, among which 45 gene-sets were up-regulated (Supplementary Figure S3a-f) whereas six gene-sets were down-regulated (Supplementary Figure S3g). Among the 3,153 proteins, 1,886 proteins were redundantly annotated in the up-regulated gene-sets that include: *response to bacterium*, *oxidative phosphorylation*, *carboxylic acid catabolic process*, *cellular component disassembly*, *mitochondrial translation*, *ion channel transport*, *monovalent inorganic cation homeostasis, protein localization* and *protein maturation* (Fig. 2, left). Interestingly, 409 proteins were annotated in the down-regulated gene-sets that include *cilium organization* and *Golgi-associated vesicle biogenesis* (Fig. 2, right), that have not been reported in relation to AF disease biology so far.

### Identification of up-regulated core biological processes and the functionally grouped network

**Figure 3:**
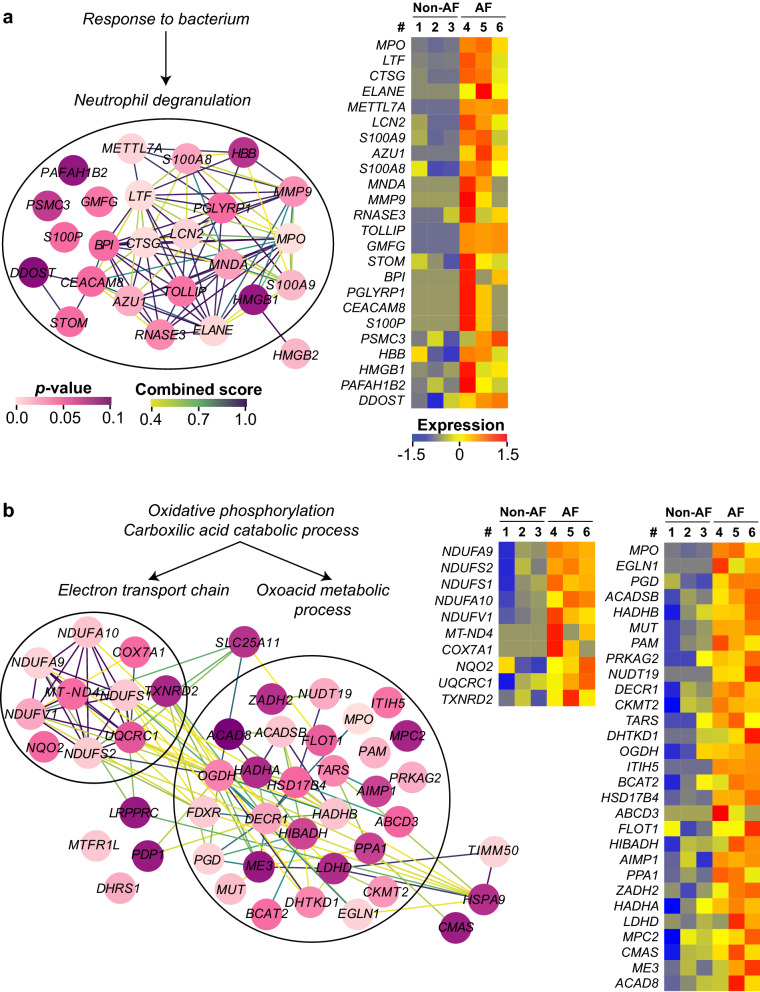
The PPI of the leading edge in the up-regulated gene-sets in AF. The PPI of each leading edge and their expression profile (heatmap) that were derived from (**a**) *Response to bacterium*, or (**b**) *Oxidative phosphorylation* and *Carboxylic acid catabolic process*. Among each gene-set, functionally grouped nodes (proteins) are encircled with its corresponding *GO terms*. Only the proteins with non-adjusted *p*-value < 0.1 are displayed here. The color of the nodes and the edges represent the non-adjusted *p*-value (0–0.1) and the STRING combined score (0.4–1.0), respectively. The color scale of expression represents the z-score of normalized spectral counts of each protein (shown as its *gene name* in each heatmap). The heatmaps were generated using R software^54^ (version 3.5.3).

Next, we focused on the proteins listed as leading edge (the core that accounts for the gene set’s enrichment signal) of each gene-set to grasp their detailed functions and their protein–protein interaction (PPI). Gene ontology enrichment analysis and STRING analysis demonstrated that the proteins listed as the leading edge of *response to bacterium* were mostly annotated to *neutrophil degranulation* (Fig. 3a). These genes demonstrated a higher fold-change and significance (Fig. 1, upper-right) than the other proteins increased in AF, and showed clear distinct expression pattern between non-AF and AF group (Fig. 3a, heatmap). The gene-sets related to oxidative phosphorylation, carboxylic acid catabolic process and protein localization had a partially overlapping leading edge that formed an extensive PPI mainly comprised of *oxoacid metabolism* and *electron transport chain* (Fig. 3b), suggesting that the metabolic process for energy generation may be enhanced in the left atrial tissue of AF patients. Of note, MPO was embedded in the PPI networks of both neutrophil degranulation and oxoacid metabolism (Fig. 3a,b) that had the biggest PPIs among all gene-sets. The leading edge in *cellular component disassembly* and *mitochondrial translation* had three functionally grouped PPI networks, which were *extracellular matrix (ECM) organization*, *autophagy* and *mitochondrial* *gene expression* (Supplementary Figure S4a). The leading edge of *ion channel transport* and *monovalent inorganic cation homeostasis* formed a weak PPI compared to other gene-sets (Supplementary Figure S4b). Small-scale PPIs of *response to oxidative stress* and *bicarbonate transport* are shown in Supplementary Figure S4c-d. There were only three proteins with p < 0.1 among the leading edge of *protein maturation* (Fig. 2) and therefore it was not further studied.

### Identification of down-regulated core biological processes and the functionally grouped network

**Figure 4:**
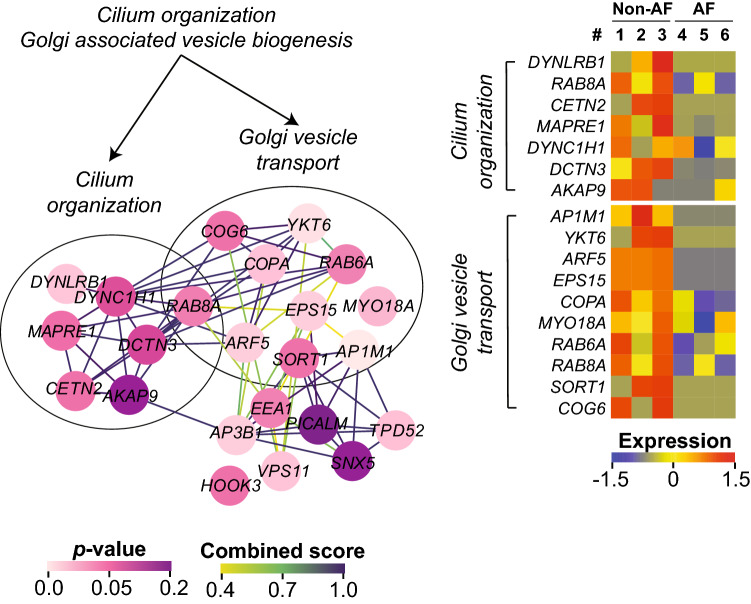
The PPI of the leading edge in the down-regulated gene-sets in AF. The PPI of the leading edge and the expression profile that were derived from *Cilium organization* and *Golgi associated vesicle biogenesis.* Only the proteins with non-adjusted *p*-value < 0.2 are displayed here. The color of the nodes and the edges corresponds to the non-adjusted *p*-value (0–0.2) and the STRING combined score (0.4–1), respectively. The color scale of expression represents the z-score of normalized spectral counts of each protein (shown as its *gene name* in each heatmap). The heatmaps were generated using R software^54^ (version 3.5.3).

The leading edge of the cilium and Golgi-associated vesicle biogenesis that were collectively down-regulated in AF formed dense PPIs that were intermingled with each other (Fig. 4). Compared to the up-regulated gene-sets, the proteins forming the down-regulated gene-sets had a weaker significance, yet a reduced abundance of these proteins in AF was distinct (Fig. 4, heatmap).

### Pivotal proteins cross-talking between over-represented biological processes in AF

**Figure 5:**
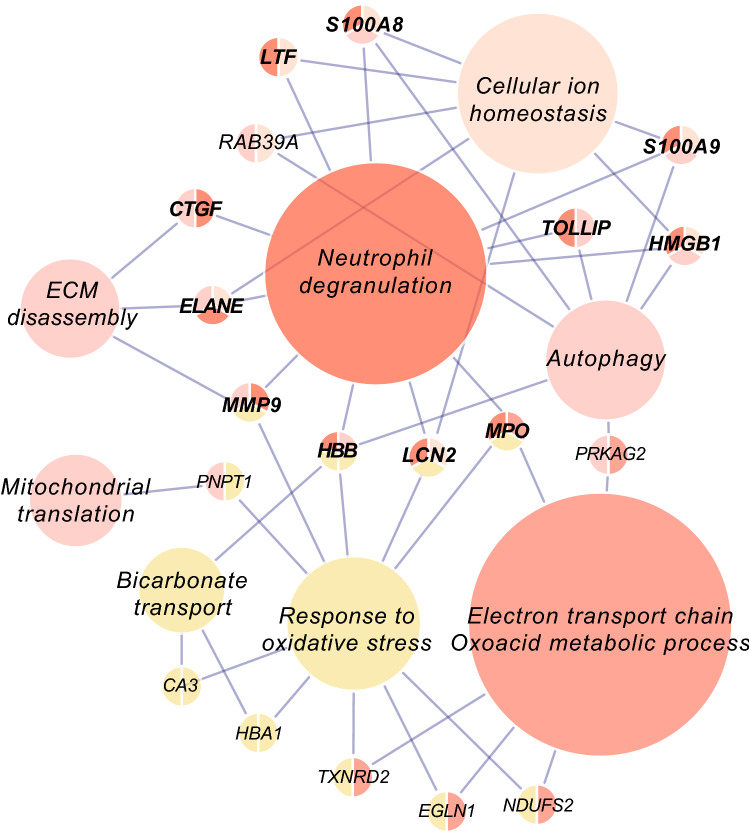
Pivotal proteins interconnecting the up-regulated biological processes in AF. The nodes represent the up-regulated biological processes in AF, and the pivotal proteins that function in multiple processes are shown as *gene names*. The size of each node represents the number of proteins annotated in it. Note that many of the proteins interconnecting the multiple biological processes are the components of neutrophil degranulation (bold lettering).

The up-regulated biological processes in AF were aligned with the proteins whose functions cross over multiple processes (Fig. 5). Remarkably, many of those proteins were the contents of neutrophil degranulation that were collectively increased in AF (Figs. 3a and 5, gene names with bold lettering). Taken together, this overview suggests that the crosstalk between over-represented biological pathways in the left atrial tissue of AF patients is for an important part mediated by neutrophil degranulation.

### Validation of up-regulated biological processes in an independent cohort

**Figure 6:**
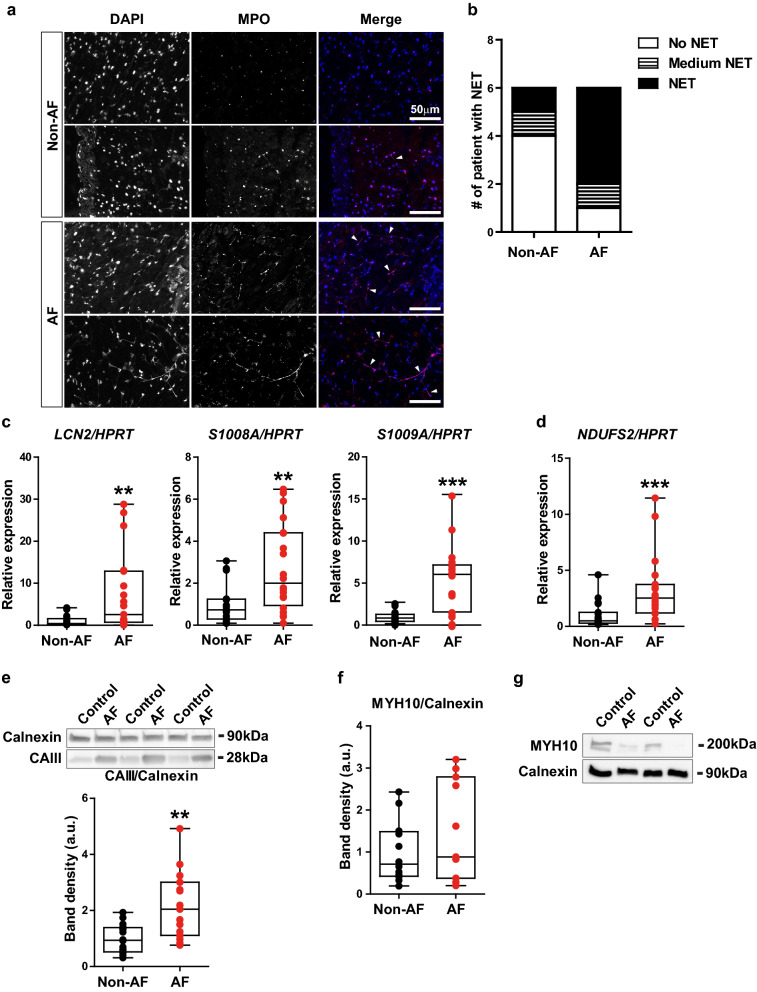
The validation of up-regulated and down-regulated biological processes in an independent cohort. (**a**) The representative fluorescent images of DAPI (the left column), MPO (middle) and merge (DAPI as blue signal and MPO as red signal) from non-AF patients (n = 2) and persistent AF patients (n = 2). Note that the fibre-like DAPI staining is co-stained with anti-MPO antibody (arrow heads in merge). Bar, 50 μm. (**b**) The number of patients whose left atrial tissue shows no NET, medium NET (localized and/or infrequent observation of NET) and NET (extensive and clear observation of NET). Non-AF: n = 6, persistent AF: n = 6. (**c**) The gene expression of *LCN2*, *S100A8* and *S100A9* in the left atrial tissue of patients. N = 18–20/group. The gene expression of *HPRT* serves as an internal control. (**d**) The gene expression of *NDUFS2* in the left atria tissue of patients. N = 19/group. (**e**) Protein levels of CAIII in the left atrial tissue of patients. The representative images of the bands are cropped from the same membrane immunoblotted with anti-Calnexin (internal control) and anti-CAIII antibody. The quantification of band density is shown below. N = 15–16/group. (**f**) Protein levels of MYH10 in the left atrial tissue of patients. (**g**) Protein levels of MYH10 in the fibroblast fraction isolated from the left atrial tissue of patients. **p < 0.01, ***p < 0.001.

To validate our proteomics analysis and data mining, we performed a validation study using an independent cohort of non-AF and AF patients (non-AF: n = 20, AF: n = 19). First, we examined if the neutrophils are present in the atrial tissue of AF patients. Cryosections of the left atrial tissues of non-AF and AF patients were immunostained with an antibody against MPO. Interestingly, we observed a fibre-like morphology of nuclei (DAPI) that was co-localized with MPO (Fig. 6a, arrowheads), a reminiscent morphology of an activated form of neutrophils called neutrophil extracellular traps (NET)^19^. The presence of NETs was more frequently observed in AF patients (Fig. 6b). Consistently, the gene expression of neutrophil gelatinase-associated lipocalin (*LCN2*), mainly expressed in the specific granules secreted from neutrophils, was significantly increased by 7.8-fold in the left atrial tissue of AF patients compared to non-AF patients (Fig. 6c). Furthermore, the gene expression of *S100A8* and *S100A9*, both components of calprotectin that comprises up to 60% of the neutrophil granulocytes^20^, was also significantly increased by 2.8-fold and 5.2-fold, respectively (Fig. 6c). These results indicate that the increased protein levels of neutrophil degranulation detected by proteomics analysis are derived from the infiltrated neutrophils into the atrial tissue, rather than from the circulating system.

NADH dehydrogenase [ubiquinone] iron-sulphur protein 2 (NDUFS2), a core subunit of the mitochondrial membrane respiratory chain, was one of the top differentially expressed proteins in AF (Supplementary Table 1), and annotated to electron transport chain (Fig. 3b). Consistent with our proteomics analysis, the gene expression of *NDUFS2* was significantly increased by 3.3-fold in AF as compared to non-AF (Fig. 6d). We also examined the protein levels of carbonic anhydrase 3 (CAIII), that was increased in AF with the second strongest significance (Supplementary Table 1), and interconnecting *bicarbonate transport* and *response to oxidative stress* (Fig. 5). As shown in Fig. 6e, the protein levels of CAIII were significantly increased by 2.2-fold in AF compared to non-AF (Fig. 6e, Supplementary Figure S5a). These data support the validity of our proteomics analysis.

### Validation of down-regulated biological process in an independent cohort

MYH10 was the top decreased protein in AF (Supplementary Table 1). The protein levels of MYH10 in the left atrial tissue were not significantly altered between non-AF and AF patients (Fig. 6f), however, a distinct reduction of its protein levels was observed in the fibroblast fraction isolated from the atrial tissue of AF patients compared to non-AF patients (Fig. 6g, Supplementary Figure S5b).

## Discussion

We performed an exploratory analysis of the AF proteome. Our data suggest that in the left atrial tissue of persistent AF patients neutrophil degranulation is a prominent biological event, the components of which may play a pivotal role interconnecting the other biological processes altered in patients with AF. Additionally, there appears to be a consistent downregulation of primary cilium assembly, restricted to atrial fibroblasts.

Many genes of *response to bacterium* were found to be significantly increased with a higher fold-change in the left atrial tissue of AF patients compared to non-AF patients. Of note, they were functionally grouped in *neutrophil degranulation* and were largely shared by other biological processes over-represented in AF. The up-regulated neutrophil degranulation in AF patients may be the consequence of different drug treatment, as the non-AF patients were treated with antiplatelet drug(s) whereas all the AF patients received NOACs (Tables 1 and 2). However, the blood leukocyte count was higher in non-AF patients than in AF patients, and the thrombocyte level was equivalent between the groups (Tables 1 and 2), suggesting that the up-regulated neutrophil degranulation in the left atrial tissue of AF patients occurs independently from the haematological effects of antiplatelet drugs or anticoagulants. The use of statins and anti-inflammatory medications such as steroids and nonsteroidal anti-inflammatory drugs (NSAIDs) may impact on neutrophilic inflammation including neutrophil degranulation^21,22^, however, there was no significance difference in the use of these medications between non-AF and AF patients. (Tables 1 and 2). In addition, there was no difference in C-reactive protein levels in plasma between non-AF and AF patients. Thus, the increased neutrophil degranulation in the left atrial tissue of persistent AF patients is a local change and AF specific, and not associated with the different drug treatment between the groups or systemic inflammation.

Neutrophils are critical inflammatory cells, not only functioning in innate immune responses to protect the host, but their elevated activation is also implicated in various cardiovascular diseases^23^ and generally considered not beneficial. In fact, high neutrophil to lymphocyte ratio has been described to be associated with new onset and recurrent AF^24^, and of thromboembolic stroke^25^. Furthermore, MPO, one of the components of neutrophil degranulation, has indeed been reported increased in atrial tissue of AF patients compared to patients without AF^26^, and its serum levels positively correlate with AF recurrence after catheter ablation of AF^27^. However, how increased neutrophil/lymphocyte ratio can be linked to AF pathology is incompletely understood. Our observation that neutrophil degranulation potentially drives or deteriorates other biological processes over-represented in AF may add to the understanding of this relation. For instance, external LCN2 induces apoptosis of cardiomyocytes in vitro and in vivo by altering intracellular iron levels^28^. Moreover, various proteinases secreted from neutrophils such as elastase (ELANE), cathepsin G (CTSG) and metalloproteinase 9 (MMP9) play an important role in ECM disassembly and tissue remodelling^29,30^, while CTSG can also process angiotensin I to angiotensin II^31,32^, whose profibrotic pathways are mediated by MPO in downstream^26^. CTSG and ELANE have additionally been described to promote blood coagulation cascade by degrading the tissue factor pathway inhibitor^33^. As such, excess of neutrophil degranulated proteins within atria of AF patients may potentially create or facilitate a milieu more prone to cardiomyocyte remodelling, fibrosis and thrombosis. Currently, we do not know if neutrophil degranulation in atrial tissue is the resultant phenomenon of quivering myocardium or the cause of AF persistency. Considering the versatile roles of neutrophil degranulation linked to pathophysiology of AF, and MPO as a prerequisite factor for structural remodelling as well as its capacity to promote electrical instability in the atria and vulnerability to AF^26^, neutrophil degranulation in atrial tissue may precede or coincide with AF onset, and subsequently alter the cardiac tissue homeostasis and eventually contribute to the tenacity of AF. A functional study will be needed to prove the causal relation between neutrophil degranulation and AF.

Metabolic process, roughly comprised of *oxoacid metabolic process* and *electron transport chain*, was another over-represented biological process in AF, where MPO was also annotated (Fig. 3b). How the external MPO plays in the cellular oxoacid metabolic process that connects to the electron transport chain is unknown. However, inflammation and oxidative stress induced by neutrophil degranulation that causes increased MPO may rapidly deplete nutrients and oxygen, resulting in a shift of energy supply/demand of the myocardium^34^. In addition, AF itself may create a hypermetabolic state^35^, and may spur on discordant regulation of energy metabolism. Further study is needed to explore a metabolic change of cardiac cells in response to inflammation and oxidative stress and to establish the link between the innate immune system and the metabolic process.

Interestingly, we found that the gene-set of cilium assembly and Golgi-associated vesicle biogenesis was significantly down-regulated in AF. Primary cilium functions as a cellular sensor to transduce the external stimulus to intracellular signalling^36^, and is actively involved in the profibrotic pathways such as TGF-β1 and Angiotensin II^37,38^. Recently, the involvement of primary cilium was also suggested in the disease-related pathological cardiac remodelling^39^. Furthermore, the epithelial cells with a dysfunctional primary cilium attract neutrophils by increasing the expression of chemo-attractants^40^. Neither *cilium organization* nor *Golgi-associated vesicle biogenesis* have been reported in AF pathophysiology before. Since Golgi-associated vesicles are essential for the assembly of primary cilium^41^, the ciliogenesis may be the major down-regulated biological process in the left atrial tissue of AF within our proteomics analysis. Importantly, we validated reduced protein levels of MYH10 in the fibroblast fraction from the left atrial tissue of AF patients. Although MYH10 has yet to be annotated to the GO term *cilium organization* (Fig. 3c), it was recently reported to be essential for the primary cilium biogenesis^42,43^. Considering that MYH10 is the top decreased protein in AF (Supplementary Table 1), it is intriguing to speculate that the cilium assembly may be suppressed in the fibroblasts in the left atrial tissue of AF patients. Our findings together with the literatures about the function of primary cilium may point at a central role of this organelle in the formation of the fibrotic substrate of AF. However, further studies are required to implicate this novel role of the primary cilium in the disease biology of AF.

The limitation of this study is the small sample size in the proteomics analysis, resulting in the potential false positive/negative candidates in our dataset. However, our approach to focus on the functionally grouped proteins rather than on a single protein allowed us to successfully detect the over-represented biological processes in AF, of which selected findings were validated in an independent cohort with the well powered sample size. Another limitation lies in the use of the patients undergoing CABG as control. Although the absence of AF in these patients was carefully confirmed by a combination of thorough history taking, assessment of all previously performed ECGs and rhythm recordings, the myocardium of patients with coronary artery disease is not pure control tissue and may have a tight connection with an ongoing inflammatory response^44^. Nevertheless, the consistent increase in components of neutrophil degranulation in AF patients compared to CABG patients, indicates that neutrophil degranulation in the left atrial tissue is a distinct aspect of AF and could potentially be more prominent when compared to healthy cardiac tissue. Considering the frequent coexistence of AF with other comorbidities including ischemic heart and valve diseases, the use of tissue from non-AF patients who nonetheless carry risk factors for the future development of AF, may clinically be more relevant for the comparison with AF patients who also have these risk factors, than comparing AF with non-diseased tissue.

In conclusion, our exploratory analysis of the entire AF proteome points at several core biological events that are involved in the pathophysiology of AF. Our findings were confirmed in an independent cohort and may provide important insights into the novel mechanism underlying the formation of the substrate for AF, which may provide novel therapeutic targets for AF treatment.

## Methods

### Patients’ enrolment and sample collection

Three male patients with persistent AF undergoing thoracoscopic AF ablation consisting of pulmonary vein isolation and additional left atrial lines were included in the proteomics analysis (hereafter, AF), and three male patients undergoing coronary artery bypass grafting (CABG) without a history of AF were used as a control cohort for the proteomics analysis (hereafter, non-AF). The left atrial tissues of non-AF patients was derived from tissue of participants in the PREDICT AF study (NCT03130985)^45^. An independent cohort of 20 patients without a history of AF and 19 patients with persistent AF was used to validate the results of proteomics analysis. The clinical characteristics of the patients enrolled in the proteomics analysis and in the validation study are summarized in Tables 1 and 2, respectively. Left atrial appendages (hereafter, left atrial tissue) were excised from patients during surgery and immediately snap-frozen in liquid nitrogen and stored at − 80 °C. All procedures including the use of human atrial tissue in this study were approved by the Medical Ethics Committee of Amsterdam University Medical Center and conducted in accordance with the principles of the Declaration of Helsinki. All patients enrolled in this study provided written informed consent.

### Sample preparation for liquid-chromatography coupled to tandem mass spectrometry

Samples were prepared as previously described^46^. Briefly, snap-frozen left atrial tissues were cryo-milled and solubilized in lithium dodecyl sulphate sample buffer 4X (Invitrogen, Cat#. NP0007) added with 100 mM DTT at the concentration of 5 mg/mL. The lysed samples were denatured by heating at 99 °C for 10 min, and the same amount of each sample was loaded on the 4–12% Bis–Tris protein gel (Invitrogen, Cat#. NP0335) to be fractionated by one-dimensional electrophoresis. The gel was fixed and stained by Coomassie brilliant blue R-250 dye (Thermo Fisher Scientific, Cat#. 20278) overnight (Supplementary Figure S1). The band density of each lane was quantified by ImageJ densitometry for later normalization of the input peptides to LC–MS/MS. The gel was then reduced in 10 mM DTT in 50 mM ammonium bicarbonate (NH_4_HCO_3_) for 1 h at 56 °C, and alkylated in 10 mg/mL iodoacetamide in 50 mM NH_4_HCO_3_ for 45 min at room temperature. Each lane of the gel was carefully sliced out and then cut into 5 pieces (Supplementary Figure S1, Fraction1-5) according to the protein ladder. Subsequently, each fraction was further diced into pieces of approximately 1 mm^3^, and incubated with 6.3 ng/mL sequencing grade modified trypsin (Promega, Cat#. V5111) in 50 mM NH_4_HCO_3_ at 25 °C overnight. The digested peptides in the gels were extracted by 1% formic acid and the concentration was united along with the band density of Coomassie brilliant blue.

### Label-free proteomics analysis by liquid-chromatography coupled to tandem mass spectrometry

The extracted peptides were separated on a U 3000 HPLC system (Thermo Fisher Scientific). A PepMap Acclaim analytical column of 75 μm inner diameter × 50 cm length (2 μm C18 particles, Thermo Fisher Scientific) was used. The injected peptides were eluted at a flow rate of 300 nl/min with 5–40% acetonitrile gradient and subsequently ionized by on-line electrospray ionization at a potential of 2 kV, and sent into a Q Exactive tandem mass spectrometer (Thermo Fisher Scientific). In MS, the ionized peptides intact masses were measured in the Orbitrap mass analyser. In each scan, the top 10 peptides with more than a charge state of 2 + were passed on to high-energy collision cell and fragmented into y and b fragment to acquire MS/MS spectra in the orbitrap.

### Peptide and protein identification

The FASTA protein sequence file of UniProtKB/Swiss-Prot human database (released in March 2017 with 42,161 entries) was used to match theoretical fragmented ions to the measured spectra using the MaxQuant^47^ search engine (version 1.5.4.1). Two missed cleavages were allowed. Peptide modification was set to cysteine carbamidomethylation, and methionine oxidation and N-terminal acetylation were added as variable modifications in the search parameters. The maximally allowed mass error for the precursor mass (MS) was 4.5 ppm and for and fragment mass (MS/MS) was 20 ppm, respectively. Both peptide and protein identifications were filtered at a false discover rate (FDR) 0.01 using the target-decoy strategy (default in MaxQuant).

### Data management and data mining

The mass spectrometry proteomics data have been deposited to the ProteomeXchange Consortium via the PRIDE^48^ partner repository with the dataset identifier PXD013230. Raw spectral counts of each identified protein were normalized by the total spectral counts of each sample. The top 20 proteins that are the most differentially expressed in AF is listed along with the significance (non-adjusted *p*-value) in Supplementary Table 1. Gene Set Enrichment Analysis (GSEA) was performed using a list of all the identified proteins pre-ranked by the significance (*p*-value) considered with biological relevance (fold-change)^17,18^. Gene Ontology (GO) enrichment analysis of the leading edge was directly performed on the platform of gene ontology consortium (http://www.geneontology.org/)^49^. The protein–protein interaction (PPI) network was assessed by STRING database^50^ (version 11.0) and the strength of each functional link was presented with a combined score. All the resultant omics data were visualized and edited on the platform of Cytoscape^51^.

### Immunohistochemistry

The snap-frozen left atrial tissues from the patients were cryo-sectioned at a thickness of 5 μm and fixed with 4% PFA for 10 min at room temperature. An antibody against myeloperoxidase (Abcam, Cat#. ab45977) was used at 1/100 dilution to detect neutrophils in the left atrial tissue, and then the sections were mounted with DAPI-containing mountant (Thermo Fisher Scientific, Cat#. P36931).

### Western blot analysis

Proteins were extracted from cryo-milled left atrial tissues using RIPA buffer added with proteinase inhibitor (Roche, Cat#. 11 836 13 001). For the protein extraction from fibroblast fraction, primary fibroblasts were enzymatically isolated from fresh left atrial tissues of non-AF (n = 2) and persistent AF patients (n = 2), and cultured approximately for two weeks to allow for a sufficient amount of proteins for analyses in DMEM (Thermo Fisher Scientific, Cat#. 41965) supplemented with 10% FBS, 1% penicillin–streptomycin and 0.1% gentamycin. When the cells reached to sub-confluency, they were solubilized in RIPA buffer supplemented with proteinase inhibitor cocktail, and the proteins were extracted. Equal amounts of protein extracts were then fractionated by electrophoresis and transferred onto nitrocellulose. Each antibody and dilution to detect the protein of interest are as follows: anti-CA3 (Santa Cruz, sc-373729) antibody at 1/200, anti-MYH10 (Santa Cruz, sc-376942) at 1/500, and anti-Calnexin (Calbiochem, Cat#. 208880) at 1/1000. The blots were then probed with a second antibody and detected by ECL primer (GE healthcare, Cat#. RPN2232). The images were captured by LAS4000 (GE Healthcare) and the signals were quantified by ImageJ densitometry.

### Real-time polymerase chain reaction

Total RNA was extracted from the cryo-milled frozen tissues using TRIzol reagent (Sigma-Aldrich, Cat#. T9424) following the manufacturer’s protocol. cDNA was synthesized from 500 ng of total RNA using Superscript II reverse transcriptase (Invitrogen, Cat#. 18064). Real-time polymerase chain reaction (RT-PCR) was performed on the LightCycler 480 (Roche) using SYBR green dye (Roche, Cat#. 04887352001) and the gene expression of interest was quantified according to linear regression analysis using LinRegPCR software^52^. The obtained values were normalized by the expression of hypoxanthine phosphoribosyltransferase 1 (*HPRT*). The sequence of primer sets used for amplification is shown as follows. *LCN2*: forward, 5′-CTACGGGAGAACCAAGGAGC-3′, reverse, 5′-CACTGGTCGATTGGGACAGG-3′. *S100A8*: forward, 5′- GCCAAGCCTAACCGCTATAAAA-3′, reverse, 5′-GTCAACATGATGCCCACGGAC-3′. *S100A9*: forward, 5′-TCGGCTTTGACAGAGTGCAA-3′, reverse, 5′- GCCCCAGCTTCACAGAGTAT-3′. *NDUFS2*: forward, 5′-TTTTGCCCATCTGGCTGGTTTG-3′, reverse, 5′-AAGTGTTCTCCTTCTGTCCCCA-3′, *HPRT*: forward, 5′-TGACACTGGCAAAACAATGCA-3′, reverse, 5′-GGTCCTTTTCACCAGCAAGCT-3’.

### Statistical analysis

The comparison of the protein’s normalized spectral counts between non-AF and AF was assessed by beta-binominal test^53^. The multiple comparisons were adjusted by Benjamini–Hochberg method at the restriction of FDR to 0.25. Due to the small sample size in proteomics analysis, none of the comparison except for MPO (myeloperoxidase) reached to the significance (Supplementary Table 1). For GSEA analysis, the FDR-adjusted *p*-value below 0.1 were regarded as over-represented gene-sets in AF. The comparison between two groups in validation experiments was analyzed by either student’s *t* test or Mann–Whitney U test according to the data distribution determined by Shapiro–Wilk test. A *p*-value < 0.05 was considered significant.

## Supplementary Information

Supplementary Information

## Data Availability

The mass spectrometry proteomics data have been deposited and are available in the ProteomeXchange Consortium via the PRIDE partner repository with the dataset identifier PXD013230.
