# Supplementary material for: Neutrophil degranulation interconnects over-represented biological processes in atrial fibrillation
Source: Sci Rep. 2021 Feb 3;11:2972. doi: 10.1038/s41598-021-82533-5 (PMC7859227; doi:10.1038/s41598-021-82533-5)

**Title page**

Neutrophil degranulation interconnects over-represented biological processes in atrial fibrillation

Makiri Kawasaki[†1], PhD, Eva R. Meulendijks[†1], MSc, Nicoline W.E. van den Berg[1], MD, Fransisca A. Nariswari[1], MSc, Jolien Neefs[1], MD, Robin Wesselink[1], MSc, Sarah W.E. Baalman[1], MD, Aldo Jongejan[2], PhD, Tim Schelfhorst[3], BSc, Sander R. Piersma[3], PhD, Thang V. Pham[3], PhD, Wim J.P. van Boven[1], MD, Antoine H.G. Driessen[1], MD, PhD, Connie R. Jimenez[3], PhD, Joris R. de Groot*[1], MD, PhD.

[1]Amsterdam UMC, University of Amsterdam, Heart Centre; Department of Clinical and Experimental Cardiology, Amsterdam Cardiovascular Sciences, Meibergdreef 9, 1105 AZ, Amsterdam, The Netherlands

[2]Amsterdam UMC, University of Amsterdam, Bioinformatics Laboratory, Department of Clinical Epidemiology, Biostatistics and Bioinformatics, Meibergdreef 9, 1105 AZ, Amsterdam, The Netherlands

[3]Amsterdam UMC, VU University Medical Centre, Cancer Centre Amsterdam, OncoProteomics Laboratory, De Boelelaan 1117, 1081 HV, Amsterdam, The Netherlands

[†]Both authors contributed equally to this study

*Corresponding author

*Supplementary Table 1. TOP 20 proteins most differentially expressed in the left atrial tissue of AF patients*

The list is sorted by the negative exponent of non-adjusted (non-adj) *p*-value (beta-binominal test). *N/A*, not applicable (not detected in non-AF samples). FC, fold-change. *NS*, not significant. (i/m)Q, Benjamini-Hochberg critical value.

*Supplementary Figure 1. The image of the gel stained with Coomassie brilliant blue*

The image of the gel after one-dimensional electrophoresis were fixated and stained with Coomassie brilliant blue overnight. L, ladder. F1-5, fraction 1-5. The dotted lines indicate the cutting line.

*Supplementary Figure 2. Principal component analysis*

**(a)** The result of principal component analysis (PCA) is shown in the dimension of PC1 (accounts for 28.6% of variance) and PC2 (24%). The blue and red dots represent non-AF patients (#1-3) and persistent AF patients (#4-6), respectively. **(b)** The distance between each patient is shown in a hierarchical unsupervised clustering. PCA was performed on R software[54] (version 3.5.3).

*Supplementary Figure 3. The result of GSEA analysis*

The nodes represent significantly up-regulated **(a-f)** and down-regulated gene-sets **(g)** in AF (FDR-adjusted *p*-value<0.1). Each node size represents the number of proteins annotated in it, and the edge connects two gene-sets that share >40% components. The nodes with the biggest size among the cluster (a collection of nodes) are named (*italic lettering*). Since *Oxidative phosphorylation* and *carboxylic acid catabolic process* belong to the same branch of *cellular metabolic process*, they were grouped together in **(b)**. Cellular component disassembly and mitochondrial translation share >40% components and therefore grouped together in **(c)**.

*Supplementary Figure 4. The small-scale PPI of the leading edge in the up-regulated gene-sets in AF*

**(a, b)** The PPI of each leading edge and their expression profile that were derived from *Cellular component disassembly* **(a)** and *Ion channel transport* and *Monovalent inorganic cation homeostasis* **(b)**. Among each gene-set, functionally grouped proteins are encircled with its corresponding *GO terms***.** Small-scale PPI of *Response to oxidative stress* **(c)** and *Bicarbonate transport* **(d)** that are arisen from the leading edge in *neutrophil degranulation* and *oxidative phosphorylation.* Only the proteins with non-adjusted *p*-value<0.1 are displayed here. The color of the nodes and the edges represent the non-adjusted *p*-value (0-0.1) and the STRING combined score (0.4-1),

respectively. The color scale of expression represents the z-score of normalized spectral counts of each protein (shown as its *gene name* in each heatmap). The heatmaps were generated using R software[54] (version 3.5.3).

Supplementary Figure 5. Full-length blots

**(a)** Full-length blots of the membrane immunoblotted with anti-Calnexin antibody (internal control) and anti-CAIII antibody. The right and left image are from the same membrane exposed for 90 seconds and 12 minutes, respectively. (b) Full-length blots of the membrane immunoblotted with anti-Calnexin antibody and anti-MYH10 antibody.

*Supplementary Table 1.*

| Gene | MW (kDa) | Sequence coverage (%) | Normalized spectral counts: Non-AF #1 | Non-AF #2 | Non-AF #3 | AF #4 | AF #5 | AF #6 | FC | $-Log_{10}$(p-value) (non-adj) | $-Log_{10}$[(i/m)Q] (FDR=0.25) | Significance |
|---|---|---|---|---|---|---|---|---|---|---|---|---|
| *MPO* | 73.9 | 42.9 | 4.8 | 2.9 | 4.0 | 38.5 | 42.2 | 27.2 | 9.2 | 4.2 | 4.1 | Significance |
| *CA3* | 29.6 | 70 | 0 | 0 | 0 | 4.2 | 3.9 | 2.1 | *N/A* | 3.0 | 3.8 | *NS* |
| *LTF* | 78.2 | 68.7 | 6.7 | 3.9 | 5.0 | 79.1 | 66.7 | 29.3 | 11.2 | 2.8 | 3.6 | *NS* |
| *CTSG* | 28.8 | 54.9 | 1.9 | 1.0 | 1.0 | 11.4 | 10.8 | 5.2 | 7.1 | 2.7 | 3.5 | *NS* |
| *COMTD1* | 28.8 | 16 | 0 | 0 | 0 | 2.1 | 3.9 | 2.1 | *N/A* | 2.7 | 3.4 | *NS* |
| *HIST1H1B* | 22.6 | 35.4 | 1.9 | 1.0 | 0 | 5.2 | 7.9 | 6.3 | 6.7 | 2.6 | 3.3 | *NS* |
| *ELANE* | 28.5 | 25.5 | 0 | 0 | 0 | 2.1 | 6.9 | 2.1 | *N/A* | 2.4 | 3.3 | *NS* |
| *METTL7A* | 28.3 | 22.1 | 0 | 0 | 0 | 2.1 | 2.0 | 2.1 | *N/A* | 2.3 | 3.2 | *NS* |
| *TIMM50* | 39.6 | 28 | 1.0 | 1.0 | 0 | 3.1 | 4.9 | 6.3 | 7.4 | 2.3 | 3.1 | *NS* |
| *NDUFA9* | 42.5 | 39.8 | 4.8 | 9.6 | 9.1 | 18.7 | 17.7 | 16.7 | 2.3 | 2.3 | 3.1 | *NS* |
| *MYH10* | 229.0 | 47.7 | 122.9 | 80.9 | 125.1 | 56.2 | 73.6 | 72.2 | -1.6 | 2.3 | 3.1 | *NS* |
| *EGLN1* | 43.7 | 11.9 | 0 | 0 | 0 | 3.1 | 1.0 | 2.1 | *N/A* | 2.2 | 3.0 | *NS* |
| *PGD* | 51.9 | 55.5 | 6.7 | 3.9 | 3.0 | 10.4 | 13.7 | 13.6 | 2.8 | 2.2 | 3.0 | *NS* |
| *LCN2* | 22.5 | 47 | 1.0 | 0.0 | 0.0 | 5.2 | 3.9 | 2.1 | 11.7 | 2.2 | 3.0 | *NS* |
| *NCAM1* | 80.3 | 27.3 | 25.0 | 23.1 | 20.2 | 16.6 | 9.8 | 15.7 | -1.6 | 2.2 | 2.9 | *NS* |
| *NDRG2* | 40.8 | 52 | 7.7 | 6.7 | 6.1 | 16.6 | 13.7 | 15.7 | 2.3 | 2.2 | 2.9 | *NS* |
| *FDXR* | 52.9 | 32.7 | 0 | 3.9 | 4.0 | 9.4 | 6.9 | 10.5 | 3.4 | 2.1 | 2.9 | *NS* |
| *CAND1* | 136.4 | 35.2 | 15.4 | 12.5 | 13.1 | 10.4 | 5.9 | 5.2 | -1.9 | 2.1 | 2.8 | *NS* |
| *NDUFS2* | 51.9 | 45.1 | 25.0 | 37.5 | 32.3 | 51.0 | 54.0 | 49.1 | 1.6 | 2.1 | 2.8 | *NS* |
| *CA4* | 35.0 | 22.1 | 0 | 0 | 1.0 | 3.1 | 4.9 | 2.1 | 10.0 | 2.0 | 2.8 | *NS* |

*Supplementary Figure 1.*

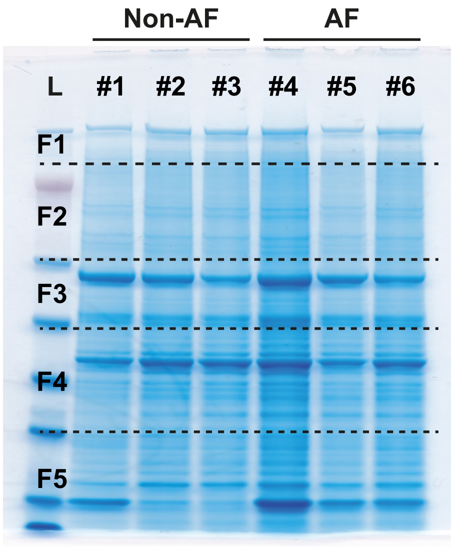

*Supplementary Figure 2.*

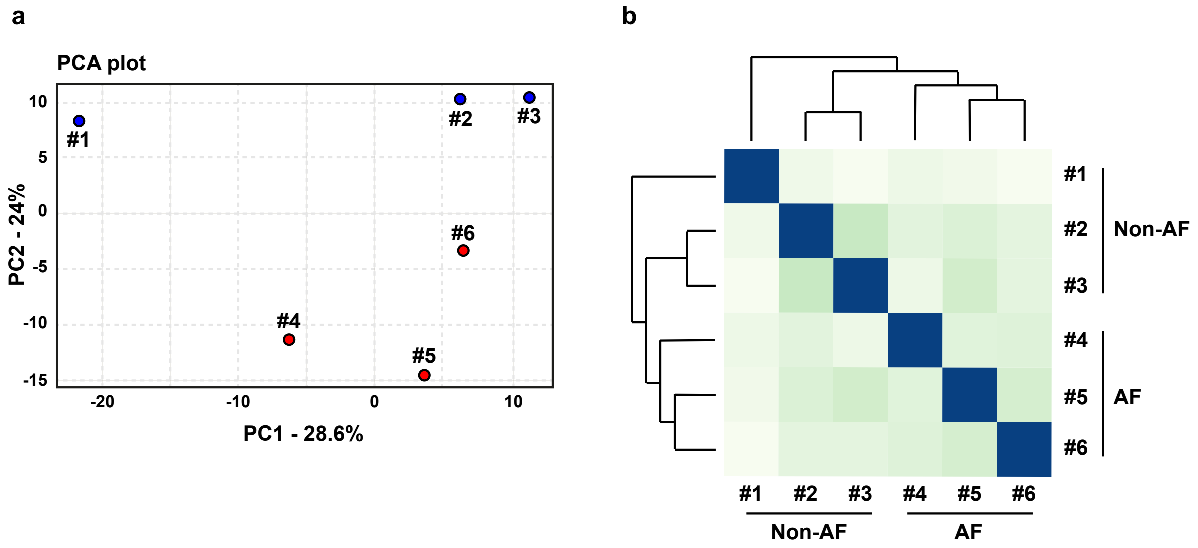

*Supplementary Figure 3.*

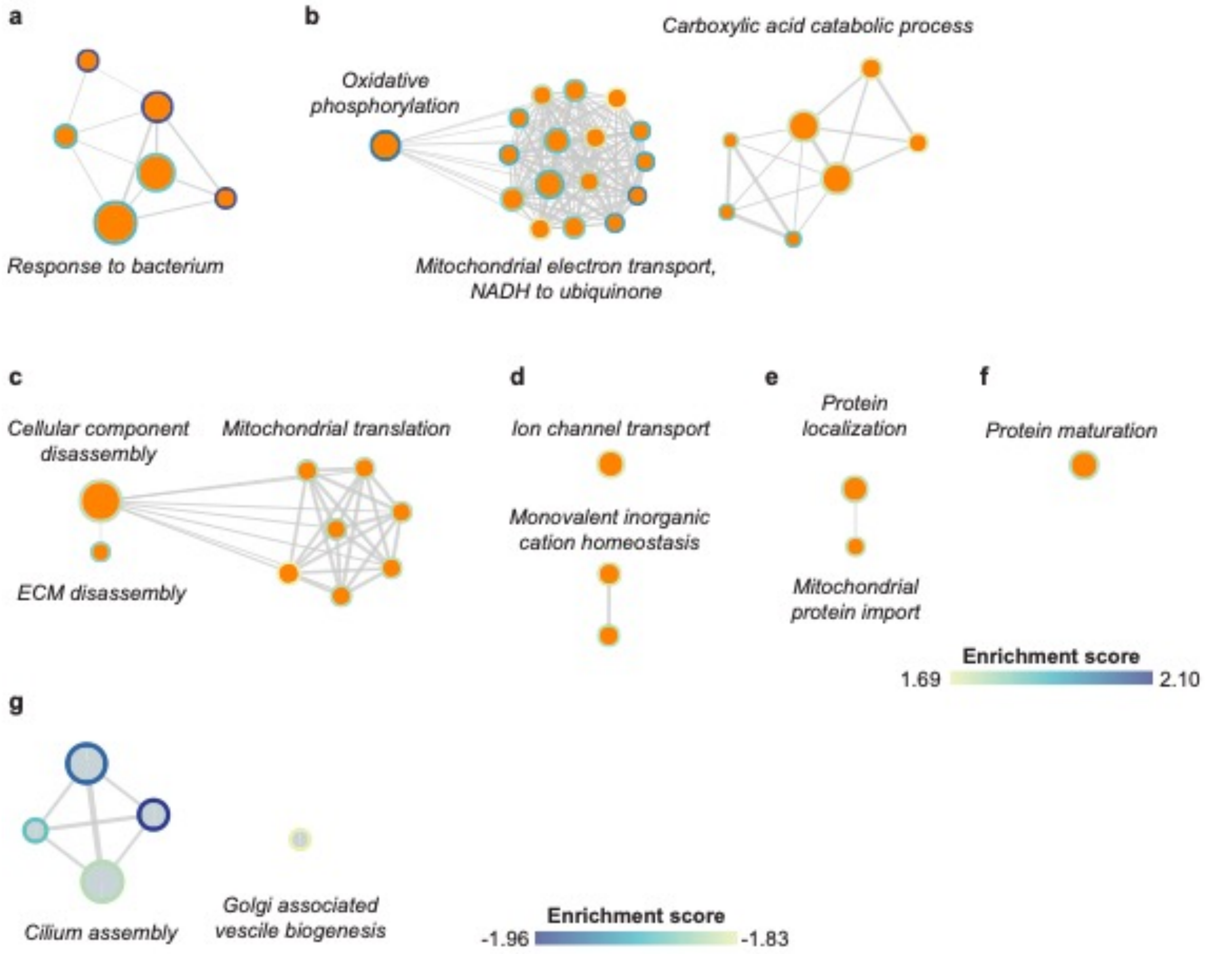

*Supplementary Figure 4.*

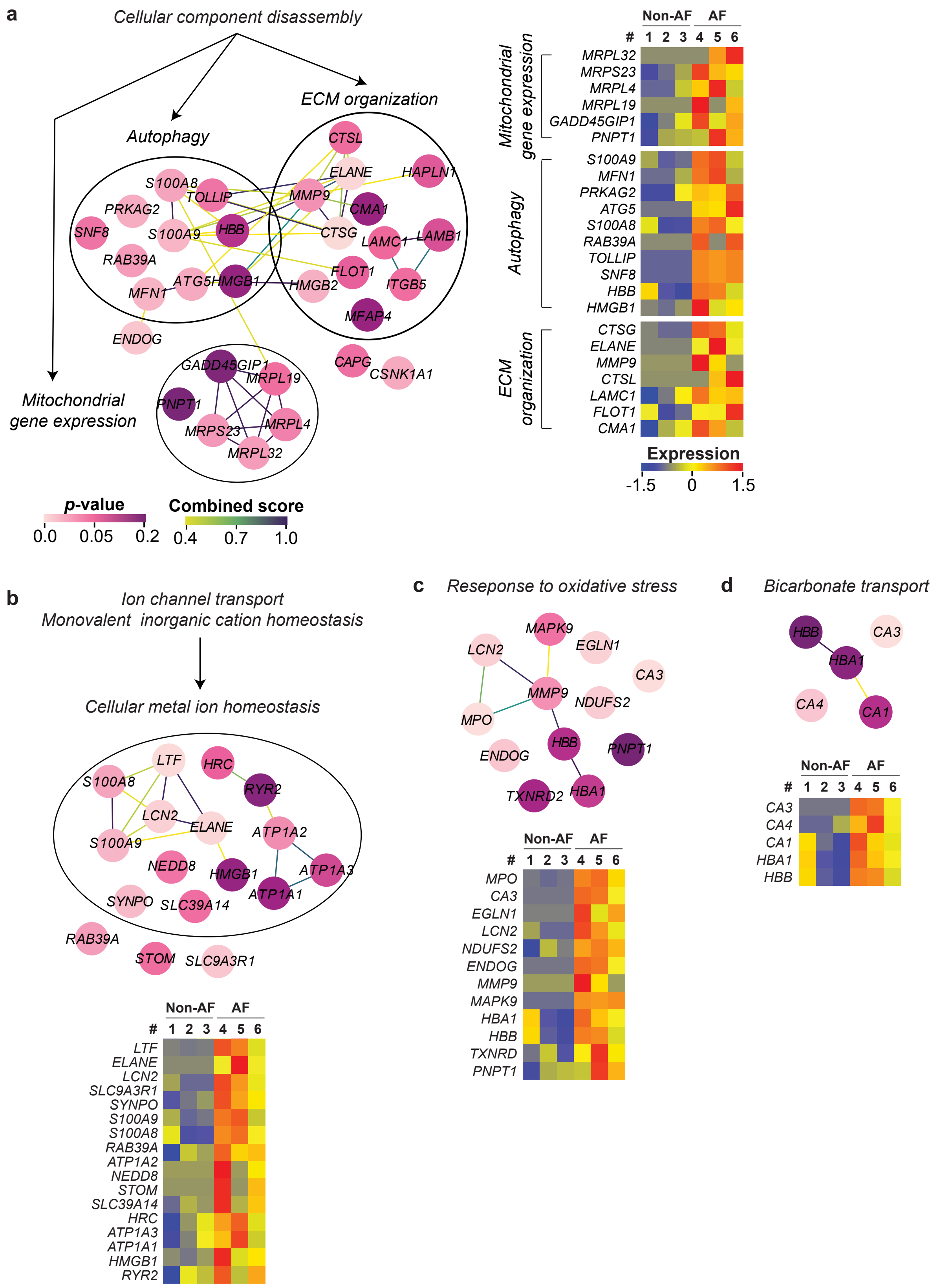

*Supplementary Figure 5.*

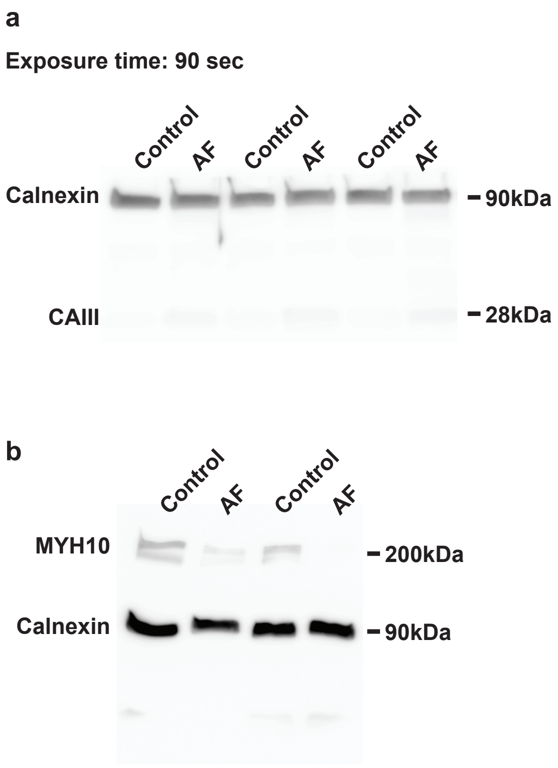

Supplement: Supplementary file 1 — Supplementary Information [file 41598_2021_82533_MOESM1_ESM.pdf]
